# Depression and Its Associated Factors among Diabetes Mellitus Patients Attending Selected Hospitals in Southwest Ethiopia: A Cross-Sectional Study

**DOI:** 10.1155/2020/6486030

**Published:** 2020-04-12

**Authors:** Adane Asefa, Ameha Zewudie, Andualem Henok, Yitagesu Mamo, Tadesse Nigussie

**Affiliations:** ^1^Department of Public Health, College of Health Science, Mizan-Tepi University, Mizan-Aman, Ethiopia; ^2^Department of Pharmacy, College of Health Science, Mizan-Tepi University, Mizan-Aman, Ethiopia

## Abstract

**Background:**

Diabetes mellitus and depression are very common diseases worldwide, and the prevalence rates of both conditions are increasing rapidly. Depression among patients with diabetes mellitus results in poor glycemic control through different mechanisms. Besides, the coexistence of a chronic medical illness with depression reduces the probability of recognizing and treating depression. The study is aimed at assessing the prevalence and factors associated with depression among adults with diabetes mellitus.

**Methods:**

A hospital-based cross-sectional study was conducted among adult diabetes mellitus patients on follow-up in Mizan-Tepi University Teaching Hospital and Tepi General Hospital. A consecutive sampling technique was employed to recruit the study participants, and data were collected through face-to-face interview and medical chart review. Depression was measured using Patient Health Questionnaire-nine (PHQ-9). Binary logistic regression analysis was done and a *p* value of less than 0.05 was used as a level of significance.

**Results:**

The prevalence of depression among study participants was 37.0% (95% CI 32.0%-42.0%). The majority (44.7%) of the patients had mild depression, while only 2% had severe depression. Being male (AOR = 1.92, 95% CI: 1.15-3.22), urban residence (AOR = 3.02, 95% CI: 1.57-5.78), single marital status (AOR = 7.72, 95% CI: 3.6-16.53), duration of diabetes mellitus 5 years and more (AOR = 2.00, 95% CI: 1.21-3.5), and having sexual dysfunction (*AOR* = 3.55, 95% CI: 2.13-5.91) were associated with increased odds of depression among diabetes mellitus patients.

**Conclusions:**

The prevalence of depression among diabetes mellitus was high. Therefore, the patients should be thoroughly screened for this comorbid condition, and the significant factors should be addressed during routine follow-up.

## 1. Introduction

Diabetes mellitus (DM) and depression are major global public health problems, and the prevalence rates of both conditions are increasing rapidly [1, 2]. It was estimated that in 2019, there are about 463 million patients with DM worldwide, and the figure is predicted to rise to 700 million by 2045 [3]. In 2017, approximately 5 million deaths among the age of 20-99 years were due to diabetes [4]. Also, a study reported that four out of five people in the world with diabetes live in low- and middle-income countries, and these countries are facing a double burden of diabetes and infectious diseases [5]. Depression is also the leading cause of mental health-related diseases and a major contributor to the overall global burden of diseases [6, 7]. At a global level, over 300 million people were estimated to suffer from depression in 2015, which was equivalent to 4.4% of the world's population [8]. The number of new cases of depression increased from 17.2 million in 1990 to 25.8 million in 2017 [9]. Over 9.5 million of global depression cases are attributable to diabetes [10]. The occurrence of depression in people with diabetes mellitus is about two to three times higher compared to the general population [11–13]. Studies have reported varying results on the prevalence of depression in diabetes patients that ranges from 5.9% to 87% [14–19]. Researchers have argued that diabetes and depression have a bidirectional relationship; depression increases the risk of developing diabetes mellitus and vice versa [20–22].

Depression causes severe health, social, and economic consequences in patients with diabetes mellitus. It negatively affects the course of DM through hormonal, neuronal, or immune system changes that directly affect the body's ability to produce or use insulin [23]. The coexistence of depression with DM also results in poor glycemic control by causing poor self-care behaviors such as lack of physical activity [24], poor adherent to low-glucose diet and medication [25], and substance use [9, 23]. Moreover, comorbid depression in DM patients is associated with anxiety, low quality of life [26], functional impairments, increased healthcare use and cost [25], increased disability, and a higher risk of death [1]. On the other hand, the course of depression in patients with diabetes is chronic and severe; even with successful treatment, up to 80% of patients with diabetes experience depression relapse [11]. Despite all these health, social, and economic implications, depression remains unrecognized and untreated in insignificant proportions of patients with diabetes [27]. Healthcare providers may not look beyond a chronic medical illness to explain nonspecific symptoms, such as fatigue or poor concentration [9].

Evidence from literature shows that the magnitude of depression and its risk factors varied across study areas, study designs, study populations, time frames, and measurement methods in previous studies [26, 28–31]. Thus, it is difficult to precisely measure the potential public health burdens and the medical care of depression in the diabetic population. Thus, an array of studies in different settings is very important. Therefore, this study is aimed at assessing the prevalence of depression and the factors associated with DM patients in Southwest Ethiopia.

## 2. Methods

### 2.1. Study Setting and Period

The study was conducted at Mizan-Tepi University Teaching Hospital (MTUTH) and Tepi General Hospital (TGH) from July 01-31, 2018, among diabetes patients. The hospitals are located in Southern Nations Nationalities and People Regional State, Southwest Ethiopia. MTUTH is found in the Mizan Aman town and provides various health services for the population that comes from its catchment area (Bench-Sheko, West-Omo, some parts of the Sheka and Kafa zones, and Gambella regional state). TGH is located in Tepi town, and it offers health services for people that come from Shaka, some parts of the Kafa and Majang zones.

### 2.2. Study Design

A hospital-based cross-sectional study was done among adult diabetes patients on follow-up.

### 2.3. Population

The source population for the study was all adult patients with diabetes who had been on antidiabetic medication at MTUTH and TGH. Those adult diabetes patients who came for follow-up treatment during data collection time and recruited for interviews were the study population.

### 2.4. Inclusion and Exclusion

Diabetic patients aged 18 years or greater who had been on antidiabetic medication were included in the study, whereas patients who were severely sick, unable to communicate, and patients with known mental disorders were excluded from the study.

### 2.5. Sample Size Determination and Sampling Procedure

The sample size was calculated using a formula for a single population proportion based on the assumption of 50% prevalence of sexual dysfunction, with a 5% margin of error and a 95% confidence interval. After adding a 10% contingency for nonresponse, the total sample size was calculated to be 423 patients. The sample was allocated proportionally to the facilities based on the total number of patients on follow-up at each facility. The patients on follow-up were identified using the medical registry book. Finally, the study participants were recruited consecutively based on their arrival at the Hospitals.

### 2.6. Variables of the Study

The outcome variable was depression. The predictor variables were sociodemographic factors (age, sex, religion, marital status, educational status, ethnicity, and residence), medical-related factors (types of DM, duration of DM, blood glucose, sexual dysfunction, and complications or comorbidities), and behavioral-related factors (physical activity, adherence to antidiabetic medication, and substance use).

### 2.7. Measurements

Data were collected using a structured questionnaire through interviewer-administered and record review methods. The tool was adapted from related literature [32, 33] and tailored to the local context. The tool was translated to a local language, “Amharic,” then back to English, to ensure its consistency. Individuals who were proficient in both languages did the translation. Independent translators did the forward and backward translations, and the expert panel resolved the discrepancy between a forward translation and the original questionnaire. The tool was pretested on 5% of the sample size before the actual data collection and modified accordingly.

Depression was assessed based on the patient's self-reported symptoms of depression within the past fourteen days of the survey using Patient Health Questionnaire-Nine (PHQ-9). The PHQ-9 has nine items that are answered on a four-point scale (0 = not at all, 1 = various days, 2 = more than half of the days, and 3 = nearly every day). The scores were summed, and the total score was categorized into 0–4, 5-9, 10-14, 15-19, and 20-27 to indicate no or minimal depression, mild depression, moderate depression, moderately severe depression, and severe depression, respectively. The overall score greater than or equal to 10 indicates the presence of depression [33].

Sexual dysfunction was evaluated by Change in Sexual Function Questionnaire (CSFQ-14), which has 14 items that are answered on five-point Likert scales. The responses were summed, and the total scores of 47 and below for males and 41 and below for females indicate the presence of sexual dysfunction [32]. The serum glucose level was measured using more recent fasting blood sugar (FBS) measurements over the past three months. The average three months FBS level ≤ 130 mg/dL was considered good glycemic control, whereas >130 mg/dL was classified as poor glycemic control. Data related to complications or comorbidities were reviewed from the medical records. Conditions such as nephropathy, retinopathy, diabetic neuropathy, diabetic foot ulcer, cardiac disease, and hypertension were considered complications or comorbidities.

Physical exercise is an engagement in moderate exercise such as slow walking or dancing to vigorous intensity exercise like fast walking or running for at least 2 days per week. We measured adherence to antidiabetic medication in the past seven days of the survey, and respondents were considered adherent if they took all antidiabetic medication as per the prescription of a physician. Substance use was measured based on respondents' self-reports on the consumption of alcohols (beer, wine, and local alcohols like ‘Areke' and ‘Tej'), tobacco, and Khat in any amount and frequency in the past 12 months.

### 2.8. Data Processing and Analysis

Data were entered into Epi data version 3.1 and exported to SPSS version 21 for analysis. Descriptive statistics were done for different variables. Bivariate binary logistic regression analyses were done, and variables that had a *p* value less than 0.20 were entered into the multivariable model. Finally, multivariable binary logistic regression analysis was performed using the backward variable selection technique. In the last model, a *p* value of < 0.05 was considered the level of significance and two-tail tests were used. Model fitness was evaluated using the Hosmer-Lemeshow test, and multicollinearity was checked by the variance inflation factor (VIF).

## 3. Results

### 3.1. Sociodemographic Characteristics

Of the total 423 patients planned for the interview, 398 were successfully addressed making a response rate of 94%. The mean age of the respondents was 41.76 (9.00) years. One hundred sixty (40.20%) were between 41 and 50 years of age, and nearly two-thirds (64.3%) of the respondents were male. Protestant Christians accounted for about one-third (35.4%) of the study participants. Out of the total study participants, only 12.3% were single. About 43.2% had attended secondary school or above. The majority of the study participants (71.4%) were urban residents. The prevalence of depression was 39.8% among males and 31.7% among females (*p* = 0.1). The singles had a higher prevalence of depression (69.4%) compared to married (27.8%) and divorced/widowed (46.5%) (*p* value *<* 0.001). Urban residents also had a higher prevalence of depression than rural residents (41.5% vs. 25.4%, *p* value = 0.003) (Table 1).

### 3.2. Behavioral and Medical-Related Factors

Of the total study participants, 79.9% were type 2 DM patients. The majority (87.9%) of the patients were in a poorly controlled glycemic range (FBS > 130 mg/dL). About 75% of the patients had been on antidiabetic medication for less than five years. Moreover, 39% of the patients had complications/comorbidities related to DM. More than two-thirds (67.6%) of the study participants were engaged in regular physical exercise. One hundred fifty-eight (39.7%) respondents had used substances in the past 12 months. More than half of the study participants (53.3%) had sexual dysfunction. The prevalence of depression was higher among patients with complications (41% vs. 32.3%, *p* < 0.001). Patients with sexual dysfunction had a higher prevalence of depression compared to those with no sexual dysfunction (48.1% vs. 24.2%, *p* value < 0.001) (Table 2).

### 3.3. Prevalence of Depression

**Figure 1:**
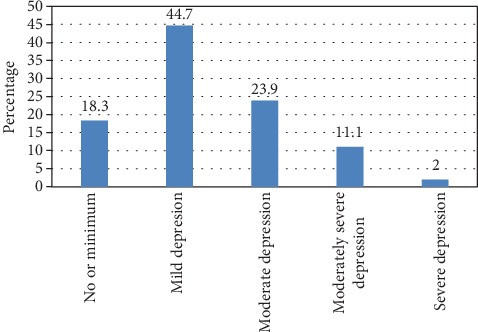
Prevalence of depression among DM patients in MTUTH and TGH, Southwest Ethiopia, July 2018 (*n* = 398).

The prevalence of depression among study participants was 37% (*n* = 398, 95% CI: 32%, 42%). The majority (44.7%) of the patients experienced mild depression, and very few (2%) had severe depression (Figure 1).

### 3.4. Factors Associated with Depression among DM Patients

During a bivariate binary logistic regression analysis, age, sex, marital status, ethnicity, educational status, residence, duration of DM, complication/comorbidities, and sexual dysfunction were found to have a *p* value less than 0.20; hence, they were included in the multivariable binary logistic regression model. However, religion, type of DM, glycemic control, physical activity, and substance use had a *p* value of greater than 0.20; therefore, they were excluded from the multivariable logistic regression analysis.

Multivariable analysis indicated that male patients had 1.92 times higher odds of depression compared to female patients (AOR = 1.92, 95% CI: 1.15-3.22). The odds of depression were 3.02 times higher among urban residents compared to rural residents (AOR = 3.02, 95% CI: 1.57-5.78). Patients with single marital status (AOR = 7.72, 95% CI: 3.60-16.53) and divorced or widowed (AOR = 1.91, 95% CI: 1.05-3.47) had higher odds of depression compared to married patients. The odds of depression among patients who had been with DM for 5 or more years were 2.05 times higher compared to patients who had been with illness for less than 5 years (AOR = 2.05, 95% CI: 1.21-3.50). Patients who had sexual dysfunction had 3.55 times higher odds of depression than those who had no sexual dysfunction (AOR = 3.55, 95% CI: 2.13-5.91) (Table 3).

## 4. Discussion

This study is aimed at assessing the prevalence of depression among DM patients on follow-up at MTUTH and TGH. It was revealed that the prevalence of depression among the study participants was 37%, and most (44.7%) had mild depression. This magnitude is almost similar to the findings of studies conducted in Malaysia (40.3%) [31], Southern India (37.5%) [34], and Nepal (34%) [35]. However, the finding is lower than studies conducted in Ethiopia, 43.6% [36], and Tanzania (87%) [17]. The inconsistency between the current and former studies might be due to variation in the tools used to measure depression, sociocultural, and behavioral-related factors among study participants.

Male patients were found to be more depressed than female patients in the current study. This finding disagrees with other studies conducted among diabetic patients [18, 26]. This inconsistency could be attributed to the differences in sociocultural, gender role, and mechanisms used to cope up with the problem between males and females across study settings. For instance, males do not disclose their problems to others and worry alone. Thus, they may not get advice or other support, which helps to relieve the problem. The current study also revealed that participants who were not in a marital union (single, divorced, or widowed) were more likely to be depressed than their counterparts (married). A similar finding was reported by a study conducted in Sri Lanka [18]. A study from southern India also indicated that being unmarried increases the risk of depression [34]. Individuals in the marital union could get social and psychological support which helps them to get relieved from depression. Supports from a partner also improve adherence to antidiabetic medications, which in turn help to prevent DM-related complications and associated depression. Similarly, evidence from the literature showed that social support has a protective effect against depression [37].

Urban residences were more likely to be depressed compared with rural residents in the current study. This might be due to differences in socioeconomic and environmental factors between the two settings. Urban residents suffer more to manage their lives compared to rural residents. This study also indicated that the odds of having depression increased with the duration of DM. A similar finding was reported by a study conducted in Saudi Arabia [37]. The increased risk of depression with the duration of the illness could be because longer duration of DM is associated with an increased risk of developing DM-related complications and comorbidities. The presence of sexual dysfunction was also found to be significantly associated with an increased risk of depression among diabetic patients in the current study. This finding agrees with the results of other studies [38, 39]. Likewise, other studies revealed an increased risk of sexual dysfunction among diabetic patients with depressive symptoms [40, 41].

### 4.1. Limitation

We employed a cross-sectional study design; therefore, we cannot be sure of the temporal relationship between the predictors and outcome variables.

## 5. Conclusions

The prevalence of depression among DM patients in the study was high. Therefore, we recommend that patients with DM should be thoroughly screened for this comorbid condition, and collaborative management for both depression and diabetes mellitus is necessary. Also, the odds of depression are higher among male patients, urban residents, single marital status, longer duration of DM (≥5 years), and patients with sexual dysfunction, and they should be addressed during routine follow-up for DM for better treatment outcome.

## Figures and Tables

**Table 1:** Sociodemographic characteristics of DM patients on follow-up in MTUTH and TGH, Southwest Ethiopia, July 2018 (*n* = 398).

Variables	*N* (%)	Depression		*χ* ^2^ test
		Yes (%)	No (%)	*p* value
Age				0.11
18-30	54 (13.6)	24 (44.4)	30 (55.6)	
31-40	117 (29.4)	37 (31.6)	80 (68.4)	
41-50	160 (40.2)	51 (31.9)	109 (68.1)	
51+	67 (16.8)	35 (52.2)	32 (47.8)	
Sex				0.10
Male	256 (64.3)	102 (39.8)	154 (60.2)	
Female	142 (35.7)	45 (31.7)	97 (68.3)	
Religion				0.22
Orthodox	136 (34.2)	47 (34.6)	89 (65.4)	
Protestants	141 (35.4)	49 (34.8)	92 (65.2)	
Muslim	96 (24.1)	37 (38.5)	59 (61.5)	
Others	25 (6.3)	14 (56.0)	11 (44.0)	
Marital status				<0.001
Married	263 (66.1)	73 (27.8)	190 (72.2)	
Single	49 (12.3)	34 (69.4)	15 (30.6)	
Divorced/widowed	86 (21.6)	40 (46.5)	46 (53.5)	
Ethnicity				0.001
Kaficho	106 (26.6)	55 (51.9)	51 (48.1)	
Amhara	101 (25.4)	36 (35.6)	65 (64.4)	
Bench	60 (15.1)	18 (30.0)	42 (70.0)	
Shakacho	50 (12.6)	9 (18.0)	41 (82.0)	
Others	80 (20.1)	29 (35.8)	52 (64.2)	
Educational status				0.090
No education	106 (26.6)	36 (34.0)	70 (66.0)	
Primary	120 (30.2)	54 (45.0)	66 (55)	
Secondary and above	172 (43.2)	57 (33.1)	115 (66.9)	
Residence				0.003
Urban	284 (71.4)	118 (41.5)	166 (58.5)	
Rural	114 (28.6)	29 (25.4)	85 (74.6)	

**Table 2:** Behavioral and medical-related factors of diabetes patients on follow-up in MTUTH and TGH, Southwest Ethiopia, July 2018 (*n* = 398).

Variables	*N* (%)	Depression		*χ* ^2^ test
		Yes (%)	No (%)	*p* value
Type of DM				0.358
T1DM	80 (20.1)	26 (32.5)	54 (67.5)	
T2DM	318 (79.9)	121 (38.1)	197 (61.9)	
Duration of DM				0.043
<5 years	299 (75.1)	102 (34.1)	197 (65.9)	
≥5 years	99 (24.9)	45 (45.5)	54 (54.5)	
Glycemic control				0.931
Poor	350 (87.9)	18 (37.5)	30 (62.5)	
Good	48 (12.1)	129 (36.9)	221 (63.1)	
Complication or comorbidity				0.07
Yes	212 (53.3)	87 (41.0)	125 (590)	
No	60 (32.3)	60 (32.3)	126 (67.7)	
Sexual dysfunction				<0.001
Yes	212 (53.3)	102 (48.1)	110 (51.9)	
No	186 (46.7)	45 (24.2)	141 (75.8)	
Substance use				0.94
Yes	158 (39.7)	58 (36.7)	100 (63.3)	
No	240 (61.3)	89 (37.1)	151 (62.9)	
Physical activity				0.557
Yes	269 (67.6)	102 (37.9)	167 (62.1)	
No	129 (32.4)	45 (34.9)	84 (65.1)	

Poor:FBS > 130 mg/dL, Good: FBS ≤ 130 mg/dL. Complications or comorbidity (nephropathy, retinopathy, diabetic neuropathy, diabetic foot ulcer, cardiac disease and/or hypertension).

**Table 3:** Factors associated with depression among DM patients on follow-up at MTUTH and TGH, Southwest Ethiopia, July 2018.

Variables	Depression		COR (95% CI)	AOR (95% CI)
	Yes (%)	No (%)		
Sex				
Male	102 (39.8)	154 (60.2)	1.43 (0.93-2.20)	1.92 (1.15-3.22)^∗^
Female	45 (32.0)	97 (68.0)	1	
Educational status				
No education	36 (34.0)	70 (66.0)	1.04 (0.62-1.73)	1.59 (0-0.82-3.12)
Primary	54 (45.0)	66 (55.0)	1.65 (1.02-2.67)	0.73 (0.39-1.35)
Secondary and above	57 (33.0)	115 (67.0)	1	
Age group				
<41	66 (35.9)	118 (64.1)	1	1
≥41	81 (37.9)	133 (62.1)	1.09 (0.72-1.64)	0.66 (0.39-1.12)
Residence				
Urban	118 (41.5)	166 (58.5)	2.08 (1.29-3.38)	3.02 (1.57-5.78)^∗^
Rural	29 (25.4)	85 (74.6)		1
Marital status				
Married	73 (27.8)	190 (72.2)	1	1
Single	34 (69.4)	15 (30.6)	5.90 (3.04-11.47)	7.72 (3.60-16.53)^∗^
Widowed and divorced	40 (46.5)	46 (55.5)	2.26 (1.37-3.74)	1.91 (1.0-3.47)
Duration of DM				
<5 years	102 (34.1)	197 (65.9)	1	
≥5 years	45 (45.5)	54 (54.5)	1.61 (1.01-2.56)	2.05 (1.21-3.50)^∗^
Sexual dysfunction				
Yes	102 (48.1)	110 (51.9)	2.51 (1.89-4.47)	3.55 (2.13-5.91)^∗^
No	45 (24.2)	141 (75.8)	1	1

^∗^ *p* value < 0.05.

## Data Availability

The data sets used and analyzed during the current study are available from the corresponding author on reasonable request.

## Abbreviations

AOR: Adjusted odds ratio
CO: Crude odds ratio
CSFQ: Change in Sexual Functioning Questionnaire
DM: Diabetes mellitus
MTUTH: Mizan-Tepi University Teaching Hospital
PHQ: Patient Health Questionnaire
SD: Sexual dysfunction
TGH: Tepi General Hospital
T1DM: Type 1 diabetes mellitus
T2DM: Type 2 diabetes mellitus
WHO: World Health Organization.
